# Trends in emergency department use by older people during the COVID-19 pandemic

**DOI:** 10.1007/s41999-021-00536-x

**Published:** 2021-07-17

**Authors:** Fergal Howley, Amanda Lavan, Eimear Connolly, Geraldine McMahon, Mustafa Mehmood, Robert Briggs

**Affiliations:** 1 Mercer’s Institute for Successful Ageing, St James’s Hospital, James’s St, Dublin 8, Ireland; 2 Department of Emergency Medicine, St James’s Hospital, James’s St, Dublin 8, Ireland; 3 Discipline of Medical Gerontology, Trinity College Dublin, Dublin 1, Ireland

**Keywords:** COVID-19, Emergency Department, Falls, Delirium

## Abstract

**Purpose:**

Reports suggest that many older people deferred seeking healthcare during the COVID-19 pandemic due to fear of contracting COVID-19. The aim of this study was to examine trends of emergency department (ED) use by older people during the first wave of the COVID-19 pandemic compared to previous years.

**Methods:**

The study site is a 1000-bed university teaching hospital with annual ED new-patient attendance of > 50,000. All ED presentations of patients aged ≥ 70 years from March to August 2020, 2019 and 2018 inclusive (*n* = 13,989) were reviewed and compared for presenting complaint, Manchester Triage Score, and admission/discharge decision.

**Results:**

There was a 16% reduction in presentations across the 6 months in 2020 compared to the average of 2018/2019. On average, 4 fewer people aged ≥ 70 years presented to the ED per day in 2020. Much of this was concentrated in March (33% fewer presentations) and April (31% fewer presentations), when the country was in ‘lockdown’, i.e. non-essential journeys were banned. There was a 20% reduction in patients presenting with stroke and cardiac complaints. In the 3 months following easing of restrictions, there was a 25% increase in falls and orthopaedic injuries when compared to 2018/2019.

**Conclusion:**

This study demonstrates a significant decline in the number of older people presenting to the ED for unscheduled care, including for potentially time-dependent illnesses such as stroke or cardiac complaints. Given the possibility of further lockdowns, it is imperative that we consider enabling strategies to ensure older people access unscheduled care in a timely manner when necessary.

## Key Summary Points

**Aim:**

To examine changing trends in presentation of older people to the emergency department during the COVID-19 pandemic compared to 2018/2019.

**Findings:**

On average 4 fewer people aged ≥70 years presented to the ED in the first 6 months of the COVID-19 pandemic (March-August 2020). There was a 20% reduction in presentations of stroke and cardiac complaints but a 25% increase in falls/injuries following easing of lockdown restrictions.

**Message:**

It is imperative that we consider enabling strategies to ensure older people access unscheduled care in a timely manner when necessary.

## Background

The emergency department (ED) is the commonest pathway by which older people access acute hospital care when unwell [1]. Currently, 20% of patients presenting to the ED are aged ≥ 65 years [2] while the number of older people presenting to EDs for unscheduled care has increased steadily in recent years against a backdrop of increased longevity generally and enhanced survival with multimorbidity and frailty [3]. Furthermore, it is projected that there will be a further marked acceleration in ED presentations in those aged ≥ 85 years in coming years [4].

Older people with COVID-19 are more likely to become unwell, to require hospitalisation and to die from the illness [5, 6]. From the early stages of the COVID-19 pandemic in Ireland, older people, as well as younger people with underlying medical illnesses, were advised to ‘cocoon’, i.e. stay indoors, and avoid face-to-face contact with other people to prevent transmission of COVID-19 within this vulnerable cohort, and to reduce pressure on emergency medical services [7]. Initial advice was to leave the house for essential reasons only. Similar strategies were applied elsewhere, with alternative terminology used such as shielding or protective isolation [8].

Following the announcement of these emergency measures, reports highlighted a significant reduction in the rate of ED presentations including in both the UK and the US [9, 10]. At the same time, a worrying pattern emerged among medical specialties of a reduction in acute presentations with complaints such as abdominal pain, chest pain or stroke, raising concerns that patients, particularly older patients most at risk from COVID-19, were delaying or avoiding seeking healthcare when unwell [11, 12]. In Ireland, the Chief Medical Officer issued a statement on 2nd April 2020 to remind the public that hospitals were open ‘for all ailments, not just COVID-19’ [13]. These concerns were supported by data suggesting 1-in-6 frail, older adults deferred seeking healthcare during the early months of the first wave [14]. As yet, no study has examined the impact of the COVID-19 pandemic on ED presentation specifically in an older cohort.

The aim of this study, therefore, was to examine the trends of ED utilisation by older people in a large urban teaching hospital during initial months of the first wave of the COVID-19 pandemic compared to similar periods in previous years. In addition, we wished to determine the impact of the COVID-19 pandemic on frailty-related presentations such as falls, delirium and stroke. Our hypothesis was, while overall numbers of presentations would be lower, that weekly and monthly trends would be influenced by, and run parallel with, public-health restrictions in place at the time, particularly those pertaining to older people and cocooning.

## Methods

This study delineates the number and nature of ED presentations in people aged ≥ 70 years during the first wave of the COVID-19 pandemic, compared to the same timepoints in 2018 and 2019, in a large urban university teaching hospital.

### Study design

The study site is a 1,000-bed university teaching hospital with an ED attendance of over 50,000 presentations per annum. St James’s Hospital has a stroke unit, and is a hub centre for cardiology, oncology, and haematology. It ran COVID-19-specific wards during the pandemic but also continued to care for non-COVID-19 illness.

All patient presentations to the ED from March to August 2020, 2019 and 2018 inclusive were reviewed retrospectively and the following information was collated for each presentation:

- Number of presentations each month
- Demographic details including age and sex
- Manchester Triage Score
- Presenting complaint based on triage note
- Admission decision
  Presenting complaint was further distilled into the following categories:
- Respiratory: breathlessness, cough, haemoptysis, or pleuritic pain
- Cardiac: chest pain, arrhythmia, palpitations
- Stroke
- Delirium
- Orthopaedic/bony injury
- Non-orthopaedic injury
- Falls: fall, faint, dizziness
- Gastrointestinal: abdominal pain, vomiting, diarrhoea, constipation, gastrointestinal bleeding
- Non-stroke neurological: seizure, headache
- Gynae/urological: flank pain, groin pain, dysuria, haematuria, urinary retention, lower abdominal pain, vaginal bleeding
- Mental health
- Eye/ear/nose/throat
- Other

Clinical categorisation of presenting complaints was based on the Hospital Inpatient Enquiry (HIPE) Code selected at triage by nursing staff when the patient presented to the ED. HIPE is the principal source of national data on discharges from acute hospitals in Ireland. Some categories we used included more than one HIPE code, for example, falls was used to code for patients with fall, faint, or dizziness HIPE code. Orthopaedic injuries were considered separately, even though the mechanism of injury could be fall-related, as their HIPE code focussed only on the injury, e.g. wrist pain. Similarly, the ‘delirium’ categorisation was based on selection of delirium or confusion HIPE code at triage, rather than on an objective assessment performed in the ED.

Each of the 6 months of interest were divided into 3 periods of at least 10 days each (with the final period of interest for each month lasting 10–11 days) to facilitate comparisons between different years. For example, March was divided into 3 separate periods from 1st to 10th, from 11 to 20th and from 21 to 31st and so on.

### Ethics

Ethical approval for this service analysis was obtained from the St James’s Hospital Research & Innovation Office (Project No 6300).

### Statistical analysis

Data were analysed using Stata version 14.1 (Stata®, College Station, TX). Data were presented descriptively. Proportions were presented with 95% confidence intervals.

A logistic regression model with admission to hospital as the dependant variable was used to assess the association with presentation during the COVID-19 pandemic. Covariates were chosen a prior based on their likelihood of association with admission to hospital and included age, sex, MTS and presenting complaint.

A *p* value ≤ 0.05 was considered statistically significant.

## Results

### Total presentations

The baseline characteristics of the study sample are outlined in Table 1. The mean age was 80 years and 54% were female.

**Table 1:** Baseline characteristics of study sample by year of presentation

	March–Aug 2020	March–Aug 2018/2019
Number of presentations (n)	4,146	4,922 (average)
Age (years), mean (95% CI) Age breakdown 70–79 years 80–89 years ≥ 90 years	79.8 (79.6 – 80.0) 0.56 (0.54 – 0.57) 0.37 (0.36 – 0.39) 0.07 (0.06 – 0.08)	80.1 (80.0 – 80.2) 0.55 (0.54 – 0.56) 0.37 (0.36 – 0.38) 0.08 (0.08 – 0.09)
Female sex (prop. with 95% CI)	0.54 (0.52 – 0.55)	0.54 (0.53 – 0.55)
MTS, mean (95% CI) MTS categories (prop. with 95% CI) Cat 1 Cat 2 Cat 3 Cat 4 Cat 5	2.75 (2.73 – 2.77) 0.02 (0.01 – 0.02) 0.34 (0.33 – 0.36) 0.52 (0.51 – 0.54) 0.11 (0.10 – 0.12) 0.00 (0.00 – 0.01)	2.70 (2.69 – 2.72) 0.02 (0.02 – 0.02) 0.39 (0.38 – 0.40) 0.47 (0.46 – 0.48) 0.12 (0.11 – 0.12) 0.01 (0.01 – 0.01)
Admitted to hospital (prop. with 95% CI)	0.57 (0.56 – 0.59)	0.55 (0.54 – 0.56)

n = number; CI = confidence interval; Prop. = proportion; MTS = Manchester Triage Score; Cat = Category

**Fig. 1:**
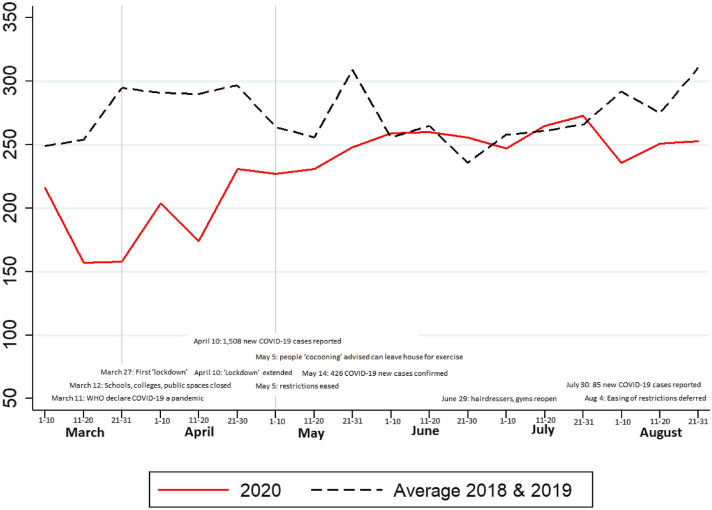
Number of presentations of patients aged ≥ 70 years to the emergency department during Wave 1 of the COVID-19 pandemic compared to 2018/2019. Data presented are absolute numbers of patients aged ≥ 70 years presenting to the emergency department during Wave 1 of the COVID-19 pandemic from March to August 2020, compared to the average number of presentations across the same months in 2018 and 2019

The trends in presentation numbers, alongside government advice regarding current COVID-19 restrictions at specific timepoints, are shown in Fig. 1 and Table 2.

**Table 2:** Trends in presentation of older people to the emergency department during the COVID-19 pandemic by presentation type

	Total	Resp.	Cardiac	Stroke	Delirium	Falls	Ortho
March–August 2020 (*n*) Average 2019/2018 (*n*) % Change in 2020 Rel. prop. 2020 Rel. prop. 2019/2018	4,146 4,922 − 16% – –	701 769 − 8% 16.9 (15.8–18.1) 15.6 (14.9–16.3)	391 491 − 20% 9.4 (8.6–10.4) 10.0 (9.4–10.6)	116 148 − 22% 2.8 (2.3–3.3) 3.0 (2.7–3.4)	140 164 − 14% 3.4 (2.9–4.0) 3.3 (3.0–3.7)	744 651 + 14% 17.9 (16.8–19.1) 13.2 (12.6–13.9)	281 335 − 16% 6.5 (5.8–7.3) 6.8 (6.3–7.3)
March 2020 (*n*) Average 2019/2018 (*n*) % Change Rel. prop. 2020 Rel. prop. 2019/2018	531 797 − 33% – –	104 180 − 42% 19.6 (16.4–23.2) 18.2 (16.4–20.2)	51 79 − 35% 9.6 (7.4 – 12.4) 9.9 (8.5–11.5)	18 22 − 14% 3.4 (2.1–5.3) 2.8 (2.1–3.7)	15 31 − 52% 2.8 (1.7–4.6) 3.8 (3.0–4.9)	116 98 + 18% 21.8 (18.5–25.6) 12.2 (10.7–13.9)	36 71 − 50% 6.8 (4.9–9.3) 8.9 (7.6–10.4)
April 2020 (*n*) Average 2019/2018 (*n*) % Change Rel. prop. 2020 Rel. prop. 2019/2018	609 877 − 31% – –	140 151 − 7% 23.0 (19.8–26.5) 17.2 (15.5–19.0)	57 103 − 45% 9.4 (7.3–12.0) 11.7 (10.3–13.3)	17 28 − 39% 2.8 (1.7–4.4) 3.2 (2.5–4.1)	19 33 − 42% 3.1 (2.0–4.8) 3.7 (2.9–4.7)	94 101 − 7% 15.4 (12.8–18.5) 11.5 (10.1–13.1)	48 56 − 14% 7.9 (6.0–10.3) 6.4 (5.3–7.6)
May 2020 (*n*) Average 2019/2018 (*n*) % Change Rel. prop. 2020 Rel. prop. 2019/2018	706 829 − 15% – –	125 127 − 2% 17.7 (15.1–20.7) 15.3 (13.7–17.1)	78 91 − 14% 11.0 (8.9–13.6) 11.0 (9.6–12.6)	20 32 − 38% 2.8 (1.8–4.4) 3.9 (3.0–4.9)	18 38 − 53% 2.5 (1.6–4.0) 4.5 (3.6–5.6)	126 128 − 2% 17.8 (15.2–20.9) 15.4 (13.8–17.3)	44 76 − 42% 6.2 (4.7–8.3) 4.6 (3.7–5.7)
June 2020 (*n*) Average 2019/2018 (*n*) % Change Rel. prop. 2020 Rel. prop. 2019/2018	775 757 + 2% – –	110 101 + 9% 14.2 (11.9–16.8) 13.3 (11.7–15.1)	82 60 + 37% 10.6 (8.6–13.0) 7.9 (6.6–9.3)	22 19 + 15% 2.8 (1.9–4.3) 2.5 (1.8–3.4)	22 20 + 10% 2.8 (1.9–4.3) 2.6 (1.9–3.5)	125 112 + 12% 16.1 (13.7–18.9) 14.8 (13.1–16.7)	55 52 + 6% 7.1 (5.5–9.1) 6.9 (5.7–8.3)
July 2020 (*n*) Average 2019/2018 (*n*) % Change Rel. prop. 2020 Rel. prop. 2019/2018	785 785 0% – –	103 123 − 16% 13.1 (10.9–15.7) 15.6 (13.9–17.5)	71 79 − 10% 9.0 (7.2–11.3) 10.0 (8.6–11.6)	22 22 0% 2.8 (1.9–4.2) 2.7 (2.0–3.7)	35 23 + 52% 4.5 (3.2–6.2) 2.9 (2.1–3.8)	152 113 + 35% 19.4 (16.7–22.3) 14.4 (12.8–16.2)	50 29 + 72% 6.4 (4.9–8.3) 3.6 (2.8–4.7)
Aug 2020 (*n*) Average 2019/2018 (*n*) % Change Rel. prop. 2020 Rel. prop. 2019/2018	740 878 − 16% – –	119 123 − 3% 16.1 (13.6–18.9) 14.0 (12.5–15.7)	52 80 − 35% 7.0 (5.4–9.1) 9.1 (7.8–10.5)	17 26 − 35% 2.3 (1.4–3.7) 2.9 (2.2–3.8)	31 21 + 48% 4.2 (3.0–5.9) 2.4 (1.8–3.2)	131 100 + 31% 17.7 (15.1–20.6) 11.3 (9.9–12.9)	52 48 + 8% 6.5 (4.9–8.5) 5.9 (4.9–7.1)

Data presented are absolute numbers of patients aged ≥ 70 years presenting to the emergency department by presenting complaint and month during Wave 1 of the COVID-19 pandemic, compared to average numbers in 2018/2019, as well as relative proportions of cases (with 95% confidence interval) related to each presenting complaint

n = number; % = percentage; rel. prop. = relative proportion of cases

On average there were 4,922 presentations of patients aged ≥ 70 years to the ED from March to August inclusive in 2018 and 2019, compared to 4,146 for the same period in 2020, representing a 16% overall reduction in acute presentations across the 6 months. The relative proportion of ED visits from patients aged ≥ 70 years did not change significantly, however, from 19.9% in 2018/2019 to 20.0% in 2020.

Much of this reduction in numbers presenting to the ED was concentrated in late March and April, when the country was in ‘lockdown’, i.e. non-essential journeys were banned. In March 2020, there were 531 presentations of people aged ≥ 70 years, an average of 17 per day, compared to an average of 797 across March 2018 and 2019 or 26 per day, a one-third reduction in the number of presentations. There was a similar reduction of 31% in the number of presentations of older people to the ED in April 2020.

On May 5th, 2020, lockdown restrictions were eased, and the public were advised that they could travel up to 5 km from their home and those who were cocooning were advised they could leave their homes for exercise. There was a 15% reduction in ED presentations in May 2020 when compared to the 2018 and 2019 figures. There was a 2% increase in the number of presentations in June 2020 compared to the average form June 2018 and 2019 and the same number of patients presented in July 2020 when compared to the average from 2018 and 2019.

After an initial opening of services such as hairdressers and gyms in late June 2020, further easing of restrictions was deferred in August due to rising case numbers. In August 2020, there was a 16% decrease in presentations from 878 in 2018/2019 (28 per day) to 740 in 2020 (24 per day).

### Presentations by illness type

**Fig. 2:**
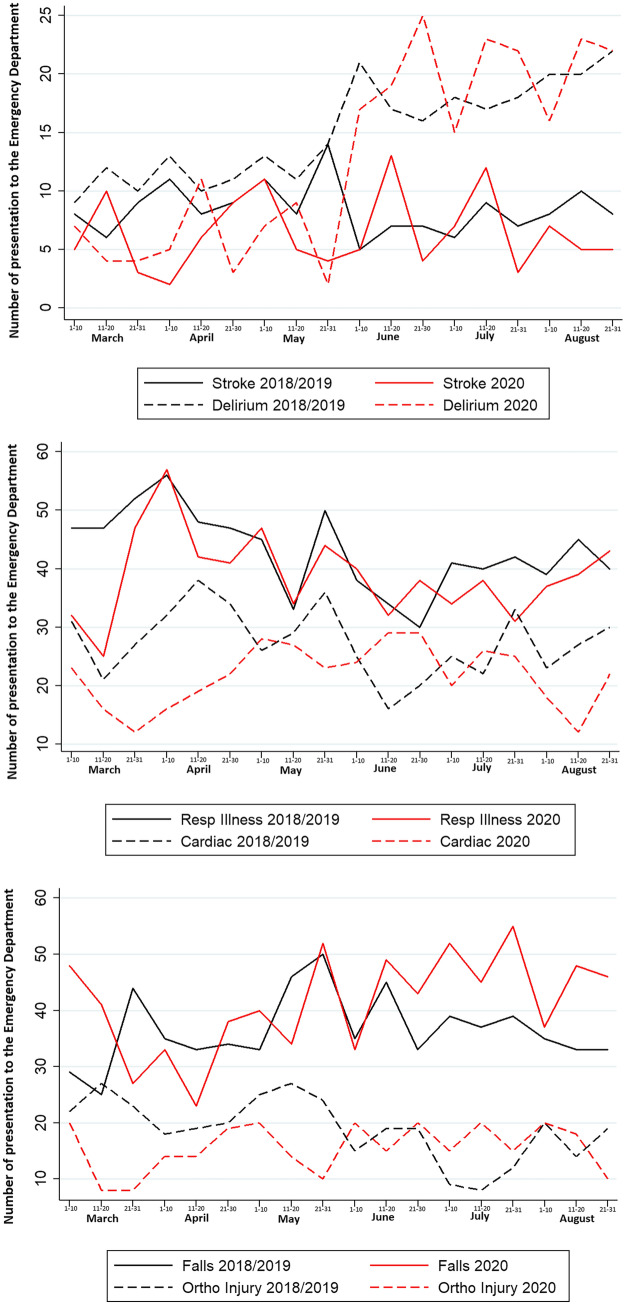
Number of presentations of patients aged ≥ 70 years to the emergency department by presenting complaint during Wave 1 of the COVID-19 pandemic compared to 2018/2019. Data presented are absolute numbers of patients aged ≥ 70 years presenting to the emergency department during Wave 1 of the COVID-19 pandemic from March to August 2020, compared to the average number of presentations across the same months in 2018 and 2019

Figure 2 and Table 2 further demonstrate the change in presentation trends for specific illnesses of interest, comparing March–August 2020 to 2018 and 2019.

Presentations with respiratory illness dropped by 8% overall in 2020 compared to prior years. After a significant initial decline in case numbers in March 2020 (by over 40%), there was a smaller difference (4% drop) in numbers across the 5 subsequent months and the relative proportion of cases comprised of respiratory illnesses remained stable.

Presentations with cardiac problems were 20% less frequent during the initial 6 months of the COVID-19 pandemic compared to the same periods in 2018 and 2019. This equated to an average of 17 fewer older people per month presenting to the ED with a cardiac-related complaint. The relative proportion of patients presenting with cardiac problems remained stable, however, at 9.4% (95% CI 8.6–10.4) in 2020 compared to 10.0% (95% CI 9.4–10.6) in 2018/2019.

There was also a 20% reduction in the numbers of older people presenting with stroke during the first 6 months of the COVID-19 pandemic, compared to 2018/2019, with a reduction of 22%, 14% and 39% in March, April and May 2020, respectively. While numbers presenting with stroke were higher in June 2020 with a 15% increase and there was no change in July 2020, there was a subsequent decline in August 2020 (15% lower).

There was an almost 50% reduction in presentations of delirium from March to May 2020 inclusive when compared to 2018/2019. In the 3 months (June–August 2020 inclusive) following the easing of cocooning restrictions, i.e. the advice that older people cocooning could go outside for exercise, there was a 25% increase in the numbers of older people presenting with delirium when compared to 2018/2019. Over 40% more older people presented with delirium from June to August 2020 than from March to April 2020.

Overall, there was a 14% increase in the number of older people presenting with falls from March to August 2020 compared to similar time periods in 2018/2019. Falls made up a significantly higher proportion of the ED cases in 2020 compared to prior years. While there a drop in presentations in April and May, there was a significant increase in numbers presenting with falls in June, July and August 2020. Similar trends were seen for orthopaedic injuries, with a decline in the early stages of the pandemic from March to May, followed by increased numbers when compared to 2018/2019 from June to August inclusive. In the 3 months following the easing of cocooning restrictions, there was a 25% increase (From an average of 454 in 2018/2019 to 565 in 2020) in falls and orthopaedic injuries involving older people presenting to the ED when compared to previous years.

### Presentation acuity and likelihood of admission

Older patients presenting from March to August 2020 were more likely to be admitted to hospital than in 208/2019, with a 57% (2383/4146) admission rate compared to 55% (2726/4922 on average) across the same months in 2018 and 2019 (X^2^ = 5.209; p = 0.022).

The mean MTS was higher in those presenting in 2020 (2.75 (95% CI 2.73–2.77) vs 2.70 (95% CI 2.69–2.72); *t* = − 3.297; *p* = 0.001) suggesting lower severity illness despite this higher admission rate.

As shown in Table 3, presentation during the COVID-19 pandemic was associated with an 18% higher likelihood of admission compared to 2018/2019 after adjusting for covariates of age, sex, MTS and presenting complaint. Other predictors of admission were male sex, age ≥ 80 years and presentation with respiratory illness, stroke, or delirium. Presenting with an orthopaedic injury and MTS > 2 was associated with lower likelihood of admission.

**Table 3:** Logistic regression model with admission to hospital as dependent variable

	Odds ratio (95% CI)	*Z*	*p*
Age range: (Ref: 70–79 years) Age 80–89 years Age ≥ 90 years	1.34 (1.24–1.46) 1.69 (1.45–1.96)	7.08 6.78	< 0.001 < 0.001
Male sex	1.09 (1.01–1.17)	2.10	0.036
Manchester Triage Score: (Ref: 3) MTS 1 MTS 2 MTS 4 MTS 5	3.54 (2.57–4.88) 4.21 (3.85–4.61) 0.21 (0.18–0.25) 0.14 (0.07–0.31)	7.74 31.18 − 19.58 − 4.89	< 0.001 < 0.001 < 0.001 < 0.001
Presenting complaint; (Ref: other PC) Respiratory Cardiac Falls Stroke Delirium Ortho injury	2.56 (2.28–2.89) 1.00 (0.87–1.15) 1.10 (0.87–1.15) 4.72 (3.36–6.62) 5.75 (4.28–7.73) 0.80 (0.68–0.94)	15.59 0.04 1.72 8.97 11.57 − 2.69	< 0.001 0.969 0.086 < 0.001 < 0.001 0.007
Presenting during COVID-19 Wave 1 (Ref: presenting at same period in 2018/2019)	1.18 (1.09–1.28)	3.87	< 0.001

Logistic regression model reporting odds ratios with 95% confidence intervals; dependent variable = admission to hospital

*ref* reference value, *MTS* Manchester Triage Score, *PC* presenting complaint, *Ortho* orthopaedic

## Discussion

This study examines ED presentations of older people during the early stages of the first wave of the COVID-19 pandemic, compared to the same period in 2018 and 2019. Overall, during these first 6 months of the pandemic there was a 16% reduction in the number of presentations to the ED with much of this due to lower numbers in March, April and early May 2020, when older people were advised to cocoon at home. Numbers began to climb back towards pre-COVID-19 levels in late May when restrictions were eased but decreased again in August 2020 after further easing of restrictions was deferred. There was no observable ‘spike’ in presentations during periods of eased restrictions within these first 6 months of the pandemic.

Overall, this equates to 4 fewer people aged ≥ 70 years presenting to the ED per day during the initial 6 months of the COVID-19 pandemic and goes against established trends of increasing numbers of frail older people presenting to acute unscheduled care in recent years. Prior studies have demonstrated significant decline in ED attendances during the COVID-19 pandemic [9, 10], including in an Irish context [13], but to our knowledge, this is the first study to examine the impact of the pandemic on the pattern and nature of ED attendance in older people.

Older people have been disproportionately affected by the COVID-19 pandemic, potentially leading to higher levels of COVID-19-related worry and concern [15]. A significant proportion of older people have deferred seeking medical attention for non-COVID-19 illnesses during the pandemic, often for fear of contracting COVID-19 [14, 16].

Different trends were seen depending on presenting complaint. While presentations with respiratory illness declined by over 40% in March 2020 compared to prior years, they were relatively similar thereafter. We have previously demonstrated, within this same cohort of frail older adults, that over 40% reported a decline in their physical health while cocooning, and almost 70% reported exercising less frequently or not exercising at all [14]. It is not surprising then that in the three months following the easing of cocooning restrictions, there was a 25% increase in falls and orthopaedic injuries compared to previous years. The significant rise in presentations of delirium, which increased by over one-quarter in this same time frame, is less expected but may be related to increased recognition of symptoms from carers or family members from whom the patient had been socially distanced up to then.

Overall, presentations with stroke and cardiac problems were one-fifth lower over the 6-month period compared to the average of 2018 and 2019, with relatively fewer numbers in each month except June, where there was an increase in both cardiac and stroke-related presentations (by 37% and 15%, respectively) as the country had a lower 7-day average number of new COVID-19 cases of 7–50 [17]. Similar trends have been seen elsewhere, with a large Stroke Centre in the US reporting a 38% fall in new stroke diagnoses during the initial 6 weeks of the COVID-19 pandemic but no change in the numbers presenting with large vessel occlusions, raising the possibility of fewer patients presenting with minor stroke symptoms [18]. A seasonal variation in terms of stroke incidence and mortality has been proposed previously and it is also possible that COVID-19-related restrictions may have reduced stroke incidence [19].

Similarly, tertiary centres have reported a decline in the number of patients presenting with myocardial infarction during the pandemic, particularly those with no-ST segment elevation myocardial infarction [20], with studies demonstrating longer times from symptom onset to presentation [21] and of patients declining hospital admission [22]. Given that further pandemic-related lockdowns are a possibility going forward, it is imperative that we deliver the message to older people that deferring medical assessment and care when unwell is unsafe and generally not in their best interests, particularly in the case of complaints such as chest pain or acute neurological symptoms.

The mean MTS for patient visits was higher in 2020 than 2018/19, suggesting lower acuity illness. There was an 18% higher likelihood of admission when presenting during the pandemic however, after adjusting for covariates including MTS. The differences between 2020 and prior years are relatively marginal, however, with similar rates of ‘Category 2’ patient visits. An older person presenting during the first wave of a global pandemic may have been viewed differently to those presenting at other times, which may explain the discrepancy in admission rates. It must also be noted that the MTS may not be an ideal tool to quantify illness severity in frail, older people [23].

There are some limitations to this study which should be noted. Data collection was retrospective and involved a single centre only. Categorisation of presenting complaint was based on the HIPE code selected at triage assessment by nursing staff for the patient visits and not on further objective measures/assessment, as it was not be feasible to incorporate further assessment information on the 14,000 patient visits included in the study. Categorisation of ‘delirium’ was based on the HIPE code selected at triage also and could therefore also include patients presenting with behavioural symptoms in the context of dementia, encephalitis, or encephalopathy. The strengths of the study include the large sample of almost 14,000 ED presentations and the fact that this is the first study to examine the impact of the first wave of the COVID-19 pandemic on ED visits by older people.

In conclusion, this study demonstrates a significant decline in the numbers of older people presenting to the ED for acute unscheduled care, including for potentially time-dependent illnesses such as stroke or cardiac problems. During periods of ‘lockdown’, presentations with falls and orthopaedic injuries declined but increased significantly beyond previous norms once restrictions were eased. Presenting to the ED remains the most frequent route by which unwell older people access acute hospital care and it is vitally important that they continue to do so in a timely manner when necessary. Given the possibility of further lockdowns and restrictions, this message needs to be communicated to older people clearly by healthcare professionals and governmental bodies to mitigate against adverse outcomes related to delayed or deferred care.

## Data Availability

Not applicable.
